# Efficacy of photobiomodulation therapy in managing iatrogenic trigeminal nerve injury: A retrospective case series

**DOI:** 10.1007/s10103-025-04709-z

**Published:** 2025-11-26

**Authors:** Farnoush Tousi, Hanna Kristine Spissøy, Hanne Molvik Larsen, Annika Rosén, Stein Atle Lie, Lado Lako Loro

**Affiliations:** 1 University of Bergen, Bergen, Norway; 2 Oral Health Centre of Expertise in Western Norway, Bergen, Norway; 3 Haukeland University Hospital, Bergen, Norway; 4 Møre and Romsdal Health Trust, Ålesund Hospital, Ålesund, Norway

**Keywords:** Photobiomodulation therapy, Trigeminal nerve injuries, Sensory disorders, Paresthesia, Retrospective case series

## Abstract

Background: Post-traumatic trigeminal neuropathy (PTTN) can significantly affect quality of life (QoL) due to pain and sensory disturbances. This study aimed to evaluate the effectiveness of photobiomodulation (PBM) therapy in patients with long-standing neurosensory disturbances associated with PTTN, using a protocol implemented at our department. Methods: A retrospective analysis was conducted on 76 cases treated between 2007 and 2018. All had neurosensory symptoms lasting more than 3 months. Data were collected from the clinical records, a structured questionnaire, and Visual Analogue Scale (VAS) scores. PBM therapy was administered using a GaAlAs diode laser. Patients were stratified based on time from injury to treatment (< 12 months vs. > 12 months). Results: Following PBM therapy, 59% of patients reported subjective improvement (30% extensive, 29% slight), while 38% reported no change and 2.6% noted slight deterioration. No statistically significant differences were observed between the groups that responded to PBM therapy and those that did not. Similarly, treatment outcomes showed no significant association with gender, etiology, affected nerve, time to treatment, or number of sessions. Conclusions: The current study did not demonstrate a statistically significant effect of PBM therapy in patients with neurosensory disturbances associated with PTTN under the applied treatment protocol. Due to the retrospective design, the absence of a control group, and the heterogeneity in patients, etiology, nerve injury types, and timing of treatment, we hypothesize that variables such as laser dosage, session intervals, and application technique may have influenced the lack of observed therapeutic benefit. Subjective improvements must be interpreted cautiously due to potential placebo effects and spontaneous nerve recovery. These findings underscore the need for standardized PBM protocols and well-designed randomized controlled trials (RCTs) to validate treatment efficacy in trigeminal nerve injuries.

## Background

 Trauma or iatrogenic injury to the trigeminal nerve (TGN) can result in neurosensory disturbances and post-traumatic trigeminal neuropathy (PTTN). These complications commonly arise following: dentoalveolar surgery, local anaesthetic injections, dental implant placement, endodontic treatment, orthognathic surgery and tumor removal in the oral and maxillofacial region [1, 2]. Among these, mandibular third molar surgery remains the leading cause of both inferior alveolar nerve (IAN) and lingual nerve (LN) injuries [3]. Reported incidence rates vary, with IAN injury ranging from 0.4 to 13.4%, and LN injury from 0 to 11% [3]. The peripheral nervous system (PNS) retains a regenerative capacity not seen in the central nervous system (CNS) [4]. Neuroplasticity and distal axonal regeneration contribute to functional recovery, although outcomes depend on injury severity (neurapraxia, axonotmesis, neurotmesis), nerve maturity, and molecular mediators such as growth factors and cytokines [5]. Neuropraxia, axonotmesis, and neurotmesis are classifications of peripheral nerve injury first described by Seddon [6] and later expanded by Sunderland [7]. These categories are fundamental for guiding both treatment strategies and prognosis [8]. Neuropraxia is the mildest form of nerve injury, typically resulting from compression. There is no disruption of the axon, although the myelin sheath may be affected. This leads to a temporary loss of nerve conduction or sensation, usually resolving within days to weeks. Axonotmesis involves disruption of the axon itself, while the surrounding myelin and connective tissue structures (such as the endoneurium, perineurium, and epineurium) may remain partially or fully intact. This results in Wallerian degeneration distal to the injury and a variable recovery period that can span months to years, depending on the extent of damage and regeneration. Neurotmesis is the most severe form, characterized by complete disruption of both the axon and its supporting connective tissue. It often results from laceration or severe crush injuries and leads to total loss of nerve conduction. While neurapraxia and axonotmesis often resolve spontaneously, neurotmesis typically requires surgical intervention [8, 9]. Persistent neurosensory disturbances have an estimated annual incidence of 18.7%, underscoring their clinical relevance [2, 10]. These deficits can significantly impair patients’ quality of life (QoL) [11, 12], and their management remains a challenge. Corticosteroids have been explored to reduce sensory impairment post-surgery, but results remain inconclusive [13, 14]. PBM therapy has gained attention as a non-invasive, well-tolerated treatment option. A 2021 systematic review confirmed its safety profile, with reversible erythema being the only reported adverse effect [15]. However, earlier studies showed inconsistent outcomes [13, 14, 16]. More recent evidence has strengthened the case for PBM. A 2023 meta-analysis of 29 studies, including 15 RCTs, concluded that PBM is effective in treating IAN injuries regardless of treatment timing [17]. A 2024 triple-blind RCT demonstrated that delayed PBM therapy still offers significant therapeutic benefit in chronic cases [18]. A 2024 systematic review highlighted PBM’s modulatory effects on ion channels and neuroinflammation, reinforcing its regenerative potential and emphasizing the need for standardized protocols [19]. Another 2024 review explored PBM combination therapies, suggesting synergistic effects when paired with pharmacological or physical interventions [20]. Despite these promising findings, variability in treatment parameters and study designs continues to limit generalizability. Standardization and long-term follow-up remain critical for clinical translation. The aim of this retrospective case series was to evaluate patients with PTTN treated with PBM therapy at the Department of Oral Surgery and Oral Medicine, University of Bergen, using a protocol originally developed by Laser Medical Systems (Denmark) in collaboration with the Universities of Nebraska, Odense University Hospital, University of Oslo, Copenhagen University Hospital, University of Aarhus, and Karolinska Hospital. However, it is important to note that the retrospective design, the absence of a control group, and heterogeneity in patient characteristics, etiology, nerve injury type, and treatment timing limit the ability to draw causal inference.

## Materials and methods

**Fig. 1:**
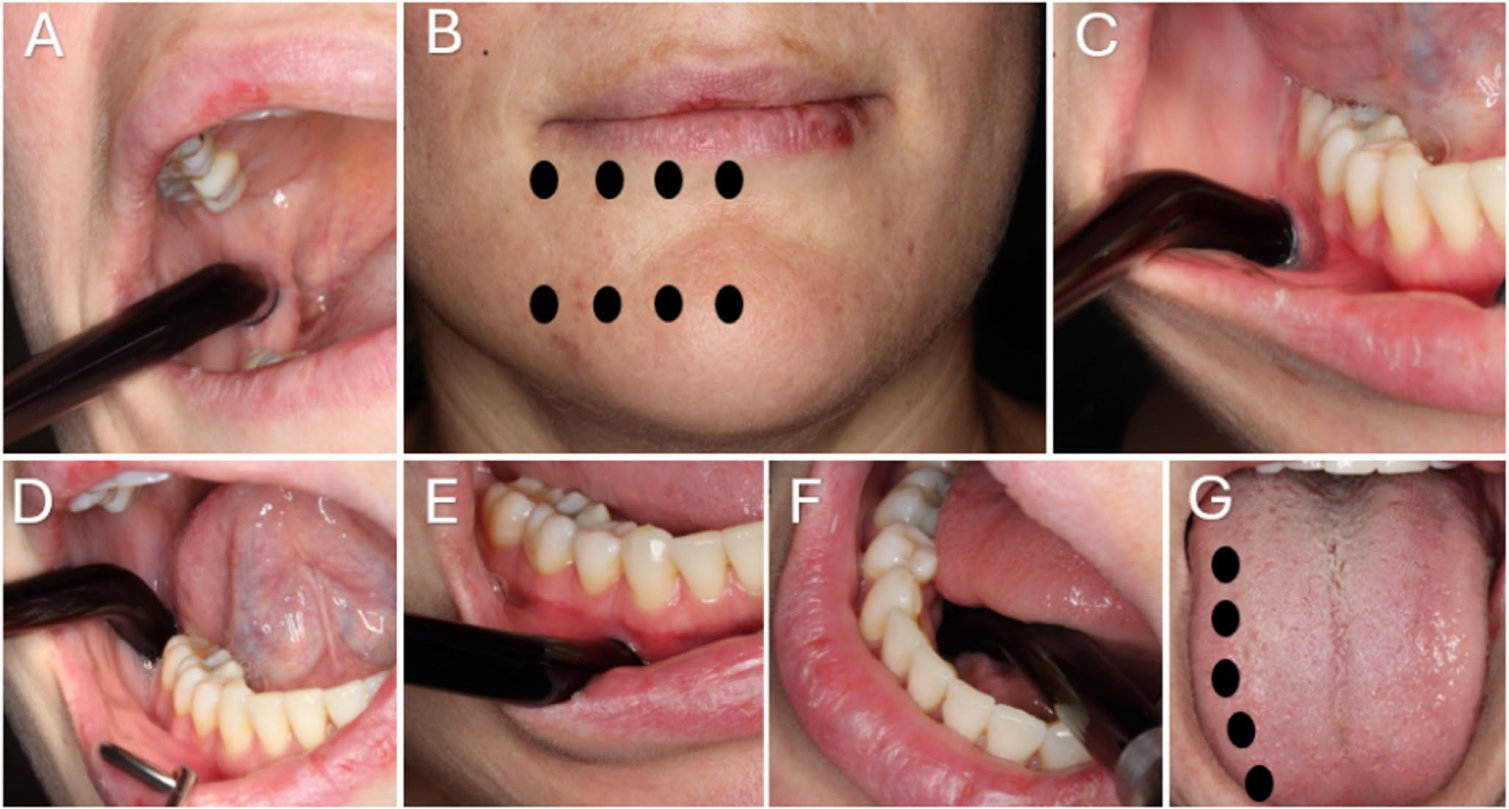
Treatment locations for inferior alveolar (**A**, **B**, **C**, **D**, **E**, **F**) and lingual nerve injury (**A**, **D**, **F**, **G**)

Patients included in this study met the following criteria: age over 18 years, symptoms associated with or classified as PTTN according to the International Classification of Orofacial Pain, 1 st edition (ICOP), 2020 [21], and the presence of long-term neurosensory disturbances lasting more than 3 months. This study was based on a convenience sample, including all eligible patients who received PBM therapy for PTTN at the Department of Clinical Dentistry, Division of Oral Surgery and Oral Medicine, University of Bergen between 2007 and 2018. A total of 119 patients were initially identified. Forty-one were excluded due to incomplete treatment or lack of follow-up, resulting in a final study population of 76 patients. A formal sample size calculation was not performed, as the study aimed to retrospectively evaluate clinical outcomes in all available cases within the defined time frame. All patients underwent a comprehensive clinical examination using a nerve injury assessment form, which included documentation of the patient’s symptoms, affected nerve, cause of nerve injury, clinical progression, and any previous treatments. Particular emphasis was placed on the patient’s subjective experience of the nerve injury, assessed using a structured questionnaire and a Visual Analogue Scale (VAS) evaluating taste, touch and pain sensations on a scale from 1 to 5, where for example: 1 = total numbness, 2 = almost no sense of touch, 3 = limited sense of touch, 4 = almost normal sense of touch, 5 = completely normal sensation. In addition, the presence of the following neurosensory symptoms was systematically recorded: hypoesthesia, paraesthesia, dysesthesia, analgesia, hyperesthesia, ageusia, anaesthesia, allodynia, and dysgeusia. Neurosensory function was further evaluated using the following clinical tests: (1) the cotton directional stroke test, (2) Contact sensibility test, (3) Needle sensibility test, (4) Two-point discrimination (5 mm and 7 mm), (5) Thermal discrimination (0–20 °C and 45–50 °C). Each patient’s record included documentation of follow-up consultations after completion of PBM therapy. PBM therapy was administered using a gallium aluminium arsenide (GaAlAs) diode laser (Photon Plus, Laser Medical Systems, Denmark) with the following specifications: 400 mW output power, 830 nm wavelength, a spot size of 0.15 cm², and a peak power density of 27,000 mW/cm². Treatment was administered intraorally (3–6 J) and/or extraorally (2–4 J), with corresponding energy densities of 13.3, 20, 26.7, 40 J/cm², depending on the energy delivered. Treatment points were distributed along the anatomical landmarks following the path of the inferior alveolar and lingual nerve (Fig. 1). All patients received an initial course of 10–15 treatment sessions. If no improvement or stagnation occurred, a second course of 10–15 sessions was offered. Follow-up evaluations were performed at 3-, 6-, and 12-month intervals after completion of therapy.

## Statistical analysis

Statistical analyses were conducted using IBM-SPSS version 29 (IBM Corp., Armonk, NY, USA). Results are presented as means with standard deviation for continuous variables, frequencies with percentages for categorical variables. Independent samples t-test was applied to compare continuous variables between groups, while Pearson’s Chi-square test and Fisher’s exact test was applied for 2 × 2 tables. For categorical variables with more than 2 categories binary logistic regression was applied. P-values less than 0.05 were considered statistically significant.

## Results

Age ranged from 19 to 78 years, with a mean age of 41.9 and a median age of 39 years. The gender distribution was 78.9% female, 21.1% male (M: F ratio = 4:15). Although the causes of nerve injury varied, surgical removal of lower third molars was the most frequent, accounting for 51.3% of cases, followed by tooth extractions 7.9%. Other etiologies are summarized in Table 1. IAN was the most frequently affected nerve, accounting for 65.8% of cases, followed by LN at 22.8%. The remaining cases involved either the buccal or infraorbital nerves (Table 1). Three patients (3.9%) presented with combined injuries to both the IAN and LN. Among all reported symptoms, the most frequent was paraesthesia (31.2%), followed by hypoesthesia (30.6%). Other symptoms such as hyperesthesia, anaesthesia, dysesthesia, allodynia, ageusia and analgesia, were observed less frequently (Table 2). The interval between injury and administration of PBM therapy ranged from four months to nearly seven years. For analytical purposes, time intervals were categorized as less than 12 months versus more than 12 months. Due to the wide variation in post-injury durations prior to treatment initiation, further subdivision into narrower intervals yielded sample sizes too small to support meaningful statistical analysis. PBM therapy was initiated within 12 months in 46 patients (60%), and after 12 months in 29 patients (39%). Three-quarters of patients received 11–15 treatment sessions. Regarding outcomes, 31 patients (40.8%) reported no noticeable improvement, including two who experienced a worsening of symptoms. Conversely, 59.2% of patients reported varying degrees of improvement after PBM therapy (Table 2). Due to the limited size of the subgroups, outcomes were categorized into two groups: “effect” (indicating slight or marked improvement) and “no effect” (indicating no change or deterioration). No statistically significant differences in PBM therapy outcomes were observed between the “effect” and “no effect” groups, or between those who received 2, 3, 4 or 6 J, male and female participants. Furthermore, treatment outcomes did not significantly vary based on etiology, affected nerve, time from injury to treatment (*p* = 0.125), or number of PBM therapy sessions (*p* = 0.763). To assess whether nerve type was associated with treatment outcome, individual p-values were calculated using binary logistic regression, with a p-value of 0.484, indicating no significant association (Table 3).

**Table 1:** Distribution of patients by gender, etiology, and affected nerve

		Frequency (*n*)	Percent (%)
Gender	Female	60	78.9
	Male	16	21.1
Etiology	Third molar surgery	39	51.3
	Tooth extraction	6	7.9
	Orthognathic surgery	5	6.6
	Anaesthetic injection	5	6.6
	Endodontic treatment/apicectomy	4	5.3
	Biopsy	4	5.3
	Trauma/fracture	4	5.3
	Bone augmentation	3	3.9
	Implant placement	2	2.6
	Cyst	2	2.6
	Suturing	1	1.3
	Genioplasty	1	1.3
Affected nerve	N. alveolaris inferior	52	65.8
	N. lingualis	18	22.8
	N. buccalis	5	6.3
	N. infraorbitalis	4	5.1

**Table 2:** Overview of symptoms, sensory disturbance/pain progression, time from nerve injury to PBM therapy initiation, PBM sessions administered, and treatment outcomes

		Frequency (*n*)	Percent (%)
Symptom	Paraesthesia	49	31.2
	Hypoesthesia	48	30.6
	Hyperesthesia	15	9.5
	Anaesthesia	10	6.4
	Dysesthesia	11	7.0
	Allodynia	11	7.0
	Ageusia	5	3.2
	Dysgeusia	5	3.2
	Analgesia	3	1.9
Sensory disturbance/pain progression	No noticeable development	30	39.5
	Improvement	26	34.2
	Deterioration	6	7.9
	Undisclosed	14	18.4
Time interval from nerve injury to initiation of PBM treatment	< 6 months	6	7.9
	6–12 months	40	52.6
	13–18 months	15	19.7
	19–24 months	2	2.6
	> 24 months	13	17.1
Number of PBM treatment	≤ 10	11	14.5
	11–15	58	76.3
	> 15	7	9.2
Treatment outcome	No noticeable improvement	29	38.2
	Slight improvement	22	28.9
	Extensive improvement	23	30.3
	Slight deterioration	2	2.6

**Table 3:** Statistical analysis of treatment outcomes

		Effect	Non effect	*P*-value	
Gender	Female	35	25	0.763^a^	
	Male	10	6		
Etiology	Extirpation	22	17	0.610^a^	
	All other etiologies	23	14		
Affected nerve	N. alveolaris inferior	32	20	0.543^a^	0.484^d^
	N. lingualis	10	8	0.718^a^	
	N. buccalis	3	2	0.673^c^	
	N. infraorbitalis	3	1	0.459^c^	
Time interval from nerve injury to initiation of PBM treatment	< 12 months	24	11	0.125^a^	
	≥ 12 months	21	20		
Number of PBM treatment	≤ 10	6	5	0.763^a^	
	11–15	34	24		
	> 15	5	2		
Mean age	(SD)	42.8 (13.1)	40.5 (14.4)	0.469^b^	

^a^ Pearson Chi-square ^b^ T-test ^c^ Fisher´s Exact test ^d^ Logistic binary regression

## Discussion

Laser dosimetry is inherently complex, involving a combination of interdependent variables, such as total energy (joules), energy density (J/cm²), irradiation time (seconds), and treatment intervals (hours to weeks), each of which can significantly influence clinical outcomes. To date, no study has systematically isolated and assessed the individual impact of these parameters. Consequently, laser settings are often chosen based on practitioner experience and subjective judgment rather than standardized, evidence-based guidelines [22].

The protocol employed in this study builds upon foundational research demonstrating promising therapeutic effects of photobiomodulation (PBM) on both peripheral and central nervous systems [23–25]. The laser parameters and application method used in this case series, aside from a 50% reduction in energy from 6 J to 3 J intraorally and from 4 J to 2 J extraorally in some cases, were originally developed for a planned multicenter study. However, none of the authors of the present study were involved in that initiative, and the outcomes of the multicenter study remain unknown to us.

To our knowledge, energy reduction was introduced in response to reports of ineffectiveness in several cases. A systematic review of PBM therapy in peripheral nerve regeneration has reported generally favorable outcomes, despite substantial variability in laser parameters. Most protocols utilized red (∼660 nm) or near-infrared (∼830–904 nm) wavelengths, with power outputs ranging from 10 to 500 mW and energy densities between 1 and 315 J/cm² [26]. In our case series, energy densities between 13 and 40 J/cm² were used, well within the range associated with beneficial effects. Nonetheless, no statistically significant improvements were observed. This suggests that energy density alone is insufficient to determine therapeutic efficacy; other factors such as wavelength, power output, treatment frequency, and delivery method are equally critical [27].

We can only speculate that variables such as cohort heterogeneity, suboptimal laser parameters, extended intervals between sessions, and the mode of application may have contributed to the lack of positive outcomes. Given the ambiguity surrounding optimal laser parameters and the absence of standardized protocols, the use of in vivo models to rigorously define treatment settings prior to clinical implementation has been proposed [28]. The ideal laser protocol for nerve regeneration should be carefully tailored to the anatomical location of the injury, with consideration given to selecting the appropriate wavelength for optimal tissue penetration, adjusting power output to prevent thermal damage, delivering energy density suited to the target tissue, and ensuring sustained stimulation over a defined therapeutic period.

Although PBM therapy, as administered in this study, did not result in statistically significant improvements in pain, sensation, or taste, over half of the participants reported slight to marked subjective improvement. These findings must be interpreted with caution due to the potential for spontaneous nerve regeneration and influence of placebo mechanisms [15]. Neurobiological pathways involving endogenous opioids, dopamine, oxytocin, and vasopressin may contribute to these effects [29–33]. Placebo-controlled trials, including one involving low-level laser therapy for temporomandibular disorder, have shown no significant difference between active and placebo groups [34].

The study showed an overrepresentation of individuals aged 30–39, likely reflecting the demographic undergoing procedures such as third molar removal or implant placement. Age is a well-documented factor in peripheral nerve recovery. Younger patients tend to have better outcomes due to enhanced neuroplasticity and regenerative capacity [5, 17]. Older patients may experience delayed or incomplete recovery, potentially due to age-related decline in axonal transport and Schwann cell responsiveness [4, 9]. Future studies should stratify outcomes by age to better understand its prognostic value and tailor interventions more effectively.

Participants varied in terms of injury mechanism, nerve injury type (neurapraxia, axonotmesis, neurotmesis), and interval from injury to treatment. Literature indicates that PBM therapy may be more effective when initiated within six months of nerve injury, aligning with the biological window for peripheral nerve regeneration [15, 35]. While some advocate delaying intervention to allow for spontaneous recovery, others emphasize the benefits of early treatment [36, 37].

This retrospective case series presents several limitations, including the use of a non-validated treatment protocol, lack of control groups and reduced sample size following stratification into homogeneous subgroups. The small size of subgroups may have resulted in underpowered statistical tests. These limitations should be considered when interpreting the findings.

It is important to note that our role was limited to retrospective analysis and interpretation of treatment outcomes in the case series, without involvement in the original study design. This distinction supports a more objective and scientifically robust evaluation by minimizing the potential for investigator bias.

## Conclusions

While numerous studies have supported the potential of PBM therapy in promoting peripheral nerve regeneration, the present study did not demonstrate a significant therapeutic effect using the applied protocol in patients with neurosensory disturbances associated with PTTN. Given the absence of a control group and the heterogeneity in patient characteristics, etiology, nerve injury type, and treatment timing, it remains speculative whether factors such as laser dosage, session intervals, and application methods contributed to the lack of positive outcomes. To accurately assess the efficacy of PBM therapy in managing trigeminal nerve injuries, future research should incorporate well-designed, prospective randomized controlled trials with standardized dosimetry, homogeneous patient cohorts, and appropriate control groups.

## Data Availability

The datasets used and analyzed during the current study are available from the corresponding author on reasonable request.

## Abbreviations

TGN: Trigeminal nerve
IAN: Inferior Alveolar Nerve
LN: Lingual Nerve
PBM: Photobiomodulation
PTTN: Post–Traumatic Trigeminal Neuropathy
RCT: Randomized Controlled Trial
QoL: Quality of Life
VAS: Visual Analogue Scale
PNS: Peripheral Nervous System
CNS: Central Nervous System
ICOP: International Classification of Orofacial Pain

## Definition of sensory disturbances

Paraesthesia: An abnormal sensation, whether spontaneous or evoked
Hypoesthesia: Decreased sensitivity to stimulation, excluding the special senses
Hyperesthesia: Increased sensitivity to stimulation, excluding the special senses
Anaesthesia: Absence of any sensation from a painful or non-painful stimulation
Analgesia: Absence of pain in response to stimulation that would normally be painful
Allodynia: Pain due to a stimulus that does not normally provoke pain
Dysesthesia: An unpleasant abnormal sensation, whether spontaneous or evoked
Ageusia: Loss of the sense of taste
Dysgeusia: Altered or impaired sense of taste
